# Gut Microbiota-Metabolome Changes in Children With Diarrhea by Diarrheagenic *E. coli*

**DOI:** 10.3389/fcimb.2020.00485

**Published:** 2020-09-18

**Authors:** Pablo Gallardo, Mariana Izquierdo, Roberto M. Vidal, Francisco Soto, Juan C. Ossa, Mauricio J. Farfan

**Affiliations:** ^1^Departamento de Pediatría y Cirugía Infantil, Facultad de Medicina, Hospital Dr. Luis Calvo Mackenna, Universidad de Chile, Santiago, Chile; ^2^Programa de Microbiología y Micología, Facultad de Medicina, Instituto de Ciencias Biomédicas, Universidad de Chile, Santiago, Chile

**Keywords:** Diarrheagenic *Escherichia coli*, microbiota, metabolome, diarrhea, children

## Abstract

**Background:** Diarrheagenic *Escherichia coli* (DEC) strains are a main cause of diarrhea worldwide in children under 5 years old. DEC virulence is strongly regulated by environmental conditions and metabolites produced by the gut microbiota in the intestinal tract. In this study, we evaluated changes in gut microbiota-metabolome in children with or without diarrhea produced by DEC pathotypes.

**Goal:** To determine gut microbiota composition and metabolome in stool samples obtained from healthy children and children with diarrhea positive for DEC pathotypes.

**Methods:** We analyzed a total of 16 age-paired stool samples: 8 diarrheal samples positive for one DEC pathotype and 8 stool samples from healthy children. To identify the microbiota composition, we sequenced the V3-V4 region of the 16S rRNA and determined operational phylogenetic units (OPU). OPU were then used to predict metabolic pathways using the PICRUSt2 software. The presence of metabolites in stool samples was determined by LC-MS. A correlation analysis was performed with the main genera from each group and main metabolites. Bacteria associated with variance of main metabolites were identified using the MIMOSA2 software.

**Results:** DEC and healthy groups showed a statistically different microbiota composition. A decrease in *Firmicutes* together with an increase in *Bacteroidetes* and *Proteobacteria* was found in the DEC group compared to the healthy group. Metabolic pathway predictions based on microbiota diversity showed that pathways involved in histidine and L-ornithine metabolism were significantly different between groups. A total of 88 metabolites detected by LC-MS were included in the metabolome analysis. We found higher levels of histamine and lower levels of ornithine in DEC samples than in the healthy group. Histamine and L-ornithine were associated with a specific microbiota species and the corresponding metabolic pathways.

**Conclusion:** Stool samples from healthy children and children positive for DEC displayed a differential metabolome and microbiota composition. A strong correlation between a gut microbiota species and certain metabolites, such as histamine and L-ornithine, was found in the DEC group. This information might be useful to identify mechanisms and signaling molecules involved in the crosstalk between microbiota and DEC pathotypes.

## Introduction

Diarrheagenic *Escherichia coli* (DEC) is the most common bacterial etiological agent of diarrhea in diverse subpopulations, both in developing and industrialized regions, and it primarily affects children under 5 years of age (Press, 2014). Generally, DEC infection involves adherence and colonization of the intestinal surface, production and secretion of virulence factors, and diarrhea, along with intestinal inflammation (Croxen et al., 2013). Even though there are data available about the regulation of DEC virulence associated with induction of an inflammatory response, information is limited to the environmental conditions in the intestine that may modulate infection. Under well-defined environmental conditions, expression of virulence genes occurs at specific sites, allowing the bacteria to initiate the infection process (Carlson-Banning and Sperandio, 2018; Alvestegui et al., 2019). Most studies have focused on unraveling the molecular mechanisms occurring inside the bacteria and little is known about the environmental factors that regulate pathogenesis at a specific time or place (Carlson-Banning and Sperandio, 2018). Therefore, an understanding of the molecular basis of DEC pathogenesis is necessary to design new strategies aimed at controlling these infections worldwide.

In recent years, gut microbiota has played a significant role in the regulation of pathogenic mechanisms. Published evidence has supported the role of specific strains from the normal gut microbiota in DEC virulence (Pacheco et al., 2012; Curtis et al., 2014; Rolhion and Chassaing, 2016). Additionally, several metabolites produced by the gut microbiota in the intestinal tract could be mediating interactions between the intestinal microbiota and enteric pathogens, such as DEC (Vogt et al., 2015). Given their abundance in the intestinal milieu, short-chain fatty acids (SCFA) have been extensively studied as signaling molecules for enteropathogens (Sun and O'riordan, 2013), including DEC, but the role of other metabolites must be explored in order to get a broader picture of virulence regulation by metabolites present in the intestinal lumen.

In this study, we determined gut microbiota and metabolome composition in stool samples obtained from healthy children and children with diarrhea positive for DEC pathotypes. Our data shows that stool samples from healthy children and children positive for DEC displayed a differential pattern of metabolites and bacterial microbiota.

## Methods

### Patients and Samples

Diarrheal and non-diarrheal stool samples were collected from September 2018 to February 2019 from patients under 5 years old treated at the Hospital Dr. Luis Calvo Mackenna (HLCM) and the HLCM daycare center, respectively, located in Santiago, Chile. All stool samples were stored at −80°C. Frozen stool samples were screened for 22 enteric pathogens using FilmArray® GI testing and selected samples were grouped as healthy (non-diarrheal, no pathogens detected) and DEC (diarrheal, with only one DEC pathotype detected). We excluded from the study children who received antibiotics, probiotics, steroidal and non-steroidal anti-inflammatory drugs 2 months prior to enrollment. Eight age-paired samples for each group were chosen for this study (Table 1).

**Table 1:** Overall microbiota findings in stool samples for DEC and healthy groups.

**Characteristics**	**DEC group**	**Healthy group**
Number of samples	8	8
Age in months (Interquartile range)	37 (27.8–48.3)	36.5 (34.3–48.5)
Pathogen detected (number of samples)	*Shigella*/EIEC (1) STEC (1) EAEC (3) EPEC (3)	
Exclusive OPUs	228	185

*DEC, Diarrheagenic E. coli*.

*EAEC, Enteroaggregative E. coli*.

*EPEC, Enteropathogenic E. coli*.

*EIEC, Enteroinvasive E. coli*.

*STEC, Shiga toxin-producing E. coli*.

### Ethics

This study was conducted in accordance with Declaration of Helsinki guidelines. The study protocol was approved by the Ethics Committee of the Universidad de Chile. Written informed consent was obtained from all parents on behalf of their children.

### DNA Extraction and Sequencing

Total DNA was extracted from each stool using the QIAamp Fast DNA Stool Mini kit (Qiagen), quantified using a Synergy HT® spectrophotometer (Biotek™) and stored at −20°C. DNA samples were shipped to Molecular Research LP (TX, USA) for DNA amplification and sequencing of the V3-V4 regions of the 16S rRNA, using the Illumina miSeq 2x300 PE.

### Microbiota Identification

Illumina raw amplicons were processed as previously described (Gallardo et al., 2017). Briefly, raw sequences were trimmed and processed using MacQiime V1.9.1-20150604, according to the default parameters for trimming (Caporaso et al., 2010). Sequences were aligned using the SINA (Pruesse et al., 2012) built-in resource on the ARB software (Ludwig et al., 2004) and OPU were assigned using the Silva132 database as the reference (Quast et al., 2013). OPU abundance was coded as an entry matrix. Data were transformed applying double square root to reduce variance between detected OPU. A redundancy analysis (RDA) and ANOVA was performed using the *Vegan* (Oksanen et al., 2019) and *ggplot2* (Wickham, 2016) packages from the RStudio 1.0.136 software. The most statistically representative genera for each group were determined using the *Indicspecies* package (De Caceres and Legendre, 2009) for R. Abundance of taxa at different levels was determined, expressing OPU abundance as percentages.

### Metabolomic Analysis

The presence of metabolites in stool samples was determined by liquid chromatography mass spectrometry (LC-MS) at MS-Omics (Denmark). Briefly, PCR grade water was added to 500 mg of stool sample, vortexed and centrifuged. Supernatants were filtered with a 0.2 μm filter and filtered supernatants were shipped to MS-Omics. A total of 156 metabolites were detected. We used the KEGG database (Kanehisa and Goto, 2000) for filtering only those with a bacterial or human origin and that were present in at least 5 of 8 samples within groups; thus, a total of 88 metabolites were selected for analysis. Metabolomic analysis was done using the *MetaboAnalystR* package (Chong and Xia, 2018) for R considering peak intensities as the input format. Data were normalized by sample median and log transformation without other data scaling. A redundancy analysis for determining metabolic structures was performed using the *vegan* package (Oksanen et al., 2019). Exploratory research on metabolites was performed using fold change analysis and a *t*-test; important features were selected by volcano plot, selecting those with a fold change of 2 or more and setting the *t*-test threshold at 0.05. Sample clustering of exploratory selected metabolites was based on their levels in samples, using hierarchical clustering, Euclidean distance and Ward algorithms of the *MetaboAnalystR* package (Chong and Xia, 2018) for R.

### Combined Analysis and Statistics

Correlation between the main genera of each group and the main metabolites detected was done using the *corrplot* package (Wei and Simko, 2017) for R. Later, a metabolic prediction was done based on the sequences of the representative OPU, using the PICRUSt2 (Douglas et al., 2020) algorithm and the PICRUSt (Langille et al., 2013) default Greengenes database as the reference (Desantis et al., 2006). Graphs were obtained with STAMP 2.9 (Parks et al., 2014), and Welch's *t*-test was used to determine significance of sequence contribution to predicted pathways. Finally, using the Web version of the MIMOSA2 (www.borensteinlab.com/software_MIMOSA2.html) package (Noecker et al., 2016) and the AGORA model, sequences from the representative OPU and metabolites selected from the prior volcano plot were used to determine a microbiota explanation for the presence of metabolite levels within the DEC groups. Representative OPUs were used to construct a metabolic model containing the metabolic reactions that each taxon is potentially capable of performing, assigning a score to each taxon-metabolite relation. Total scores were compared to metabolite measurements across all samples, and a regression analysis to assess whether scores were positively or negatively predictive of the observed metabolite levels in the samples was done. Finally, decomposing the model allowed us to identify the individual taxon contributions. The sum of the contributions of all listed taxa is equal to the unadjusted R-squared of the regression model used to predict each metabolite.

## Results

### Metabolic Pathway Predictions Using Microbiota Composition in Diarrheal and Non-diarrheal Stool Samples

**Figure 1:**
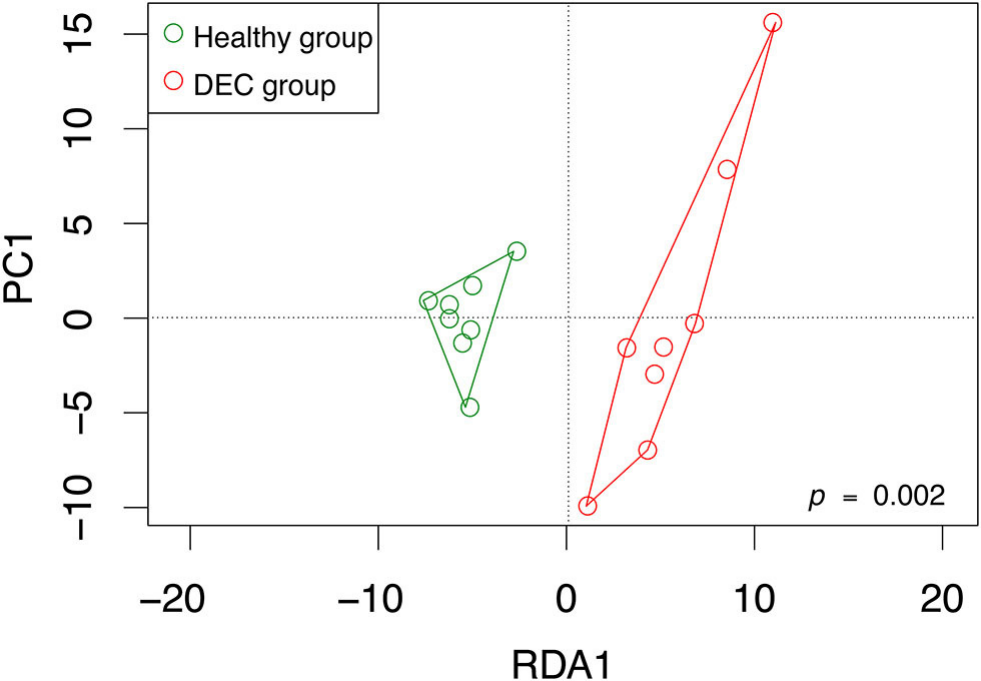
OPU community structure of healthy and DEC-positive stool samples. Microbiota community distribution of each sample and its clustering according to the sample group were analyzed by redundancy analysis. Green and red circles represent microbiota compositions found in healthy and DEC-positive stool samples, respectively. The analysis was conducted using a sample classification as the explanatory matrix and relative OPU abundance as the response matrix. Data were normalized with a double square root transformation. Clustering significance was analyzed by ANOVA, using the *vegan* package for R.

We identified a total of 755 OPU within 16 stool samples. The redundancy analysis showed that microbiota composition was statistically different among DEC and healthy groups, with a distinctive community structure clustering (Figure 1; *p* = 0.002). At *phylum* level, the DEC group presented a decreasing number of Firmicutes (81.8 ± 3.6% vs. 68.3 ± 7.5%, *p* < 0.001), as well as an increasing number of Proteobacteria (4.1 ± 1.5% vs. 11.6 ± 6%, *p* = 0.009) compared to the healthy group. We also found differences in the proportion of Bacteroidetes (7.1 ± 3.9% vs. 12.7 ± 6.7%, *p* = 0.06). All the taxa comparisons are shown in Supplementary Figure 1.

**Figure 2:**
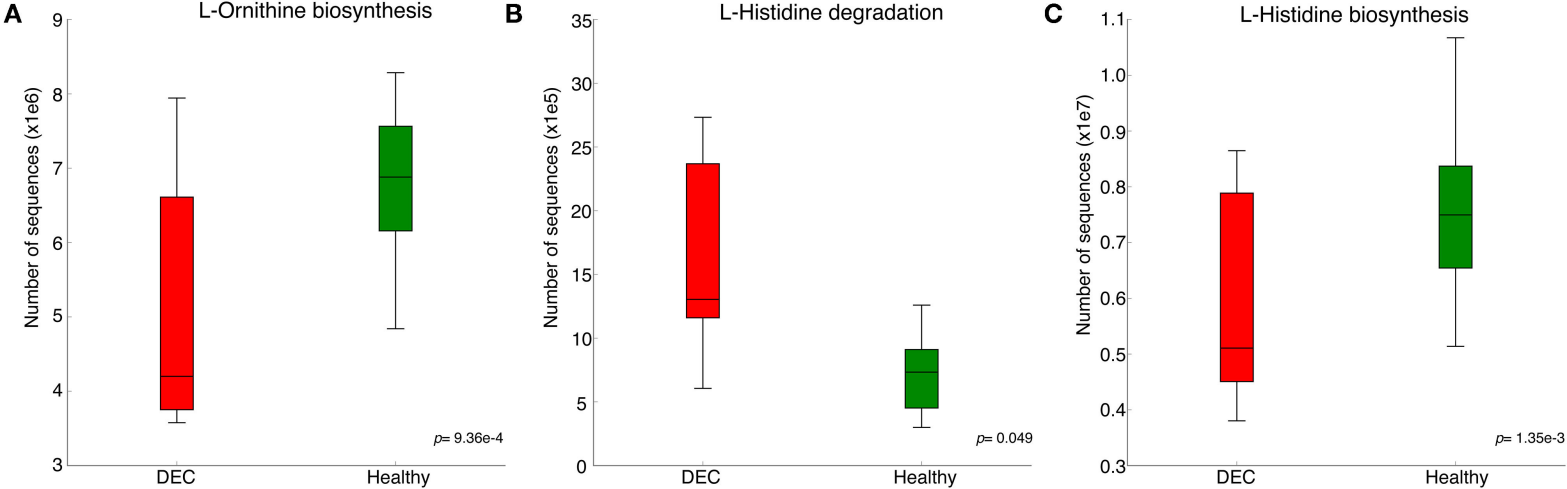
Predicted metabolic pathways related to the gut microbiota composition. Principal pathway prediction by PICRUSt2 algorithm using representative sequences of main OPUs from each group and the Greengenes database as the reference. Graphs of predictions and significance values were obtained using STAMP v. 2.1.3. Graphs represent the number of sequences in microbiota that have known genes involved in L-ornithine biosynthesis **(A)**, L-histidine degradation **(B)**, and L-histidine biosynthesis **(C)**. Box plots denote the top quartile, median, and bottom quartile, analyzed using Welch's *t*-test.

Representative sequences of each OPU were used to predict metabolic pathways that could be related to the gut microbiota composition. Pathways involved in L-histidine degradation presented a higher representation of sequences associated with DEC groups compared to healthy groups. By contrast, L-ornithine and L-histidine biosynthesis pathways were less represented in DEC groups compared to healthy groups (Figure 2). All metabolic predictions are shown in Supplementary Figure 2.

### DEC Group Displayed a Distinctive Metabolome Composition Compared to Healthy Group

**Figure 3:**
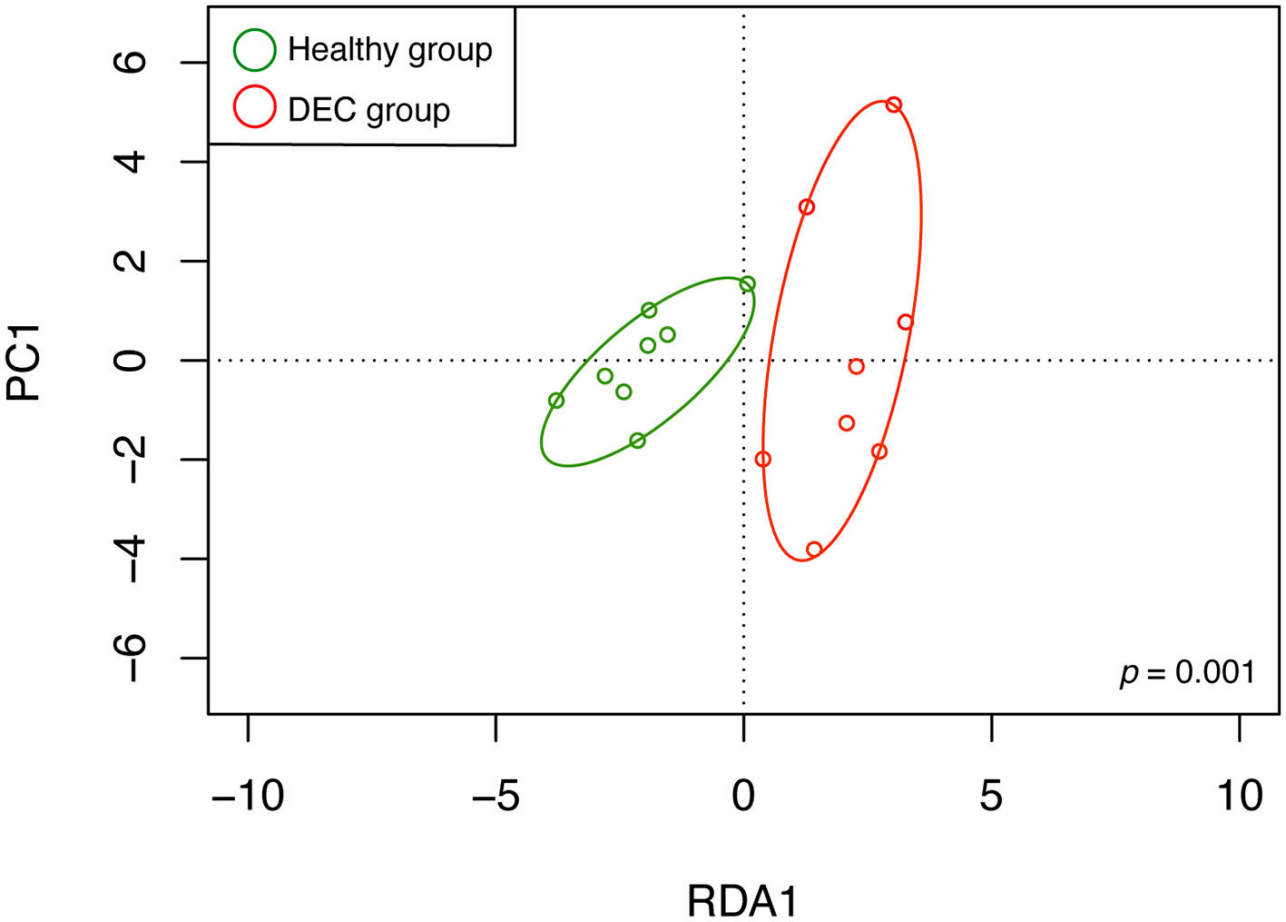
Metabolomic structure of healthy and DEC-positive stool samples. Metabolomic structure of each sample and its organization according to the sample group were analyzed by redundancy analysis. Green and red circles represent metabolome compositions found in healthy and DEC-positive stool samples, respectively. The analysis was conducted using peak intensities of 88 LC-MS-detected metabolites from stool samples. Clustering significance was analyzed by ANOVA, using the *vegan* package for R.

**Figure 4:**
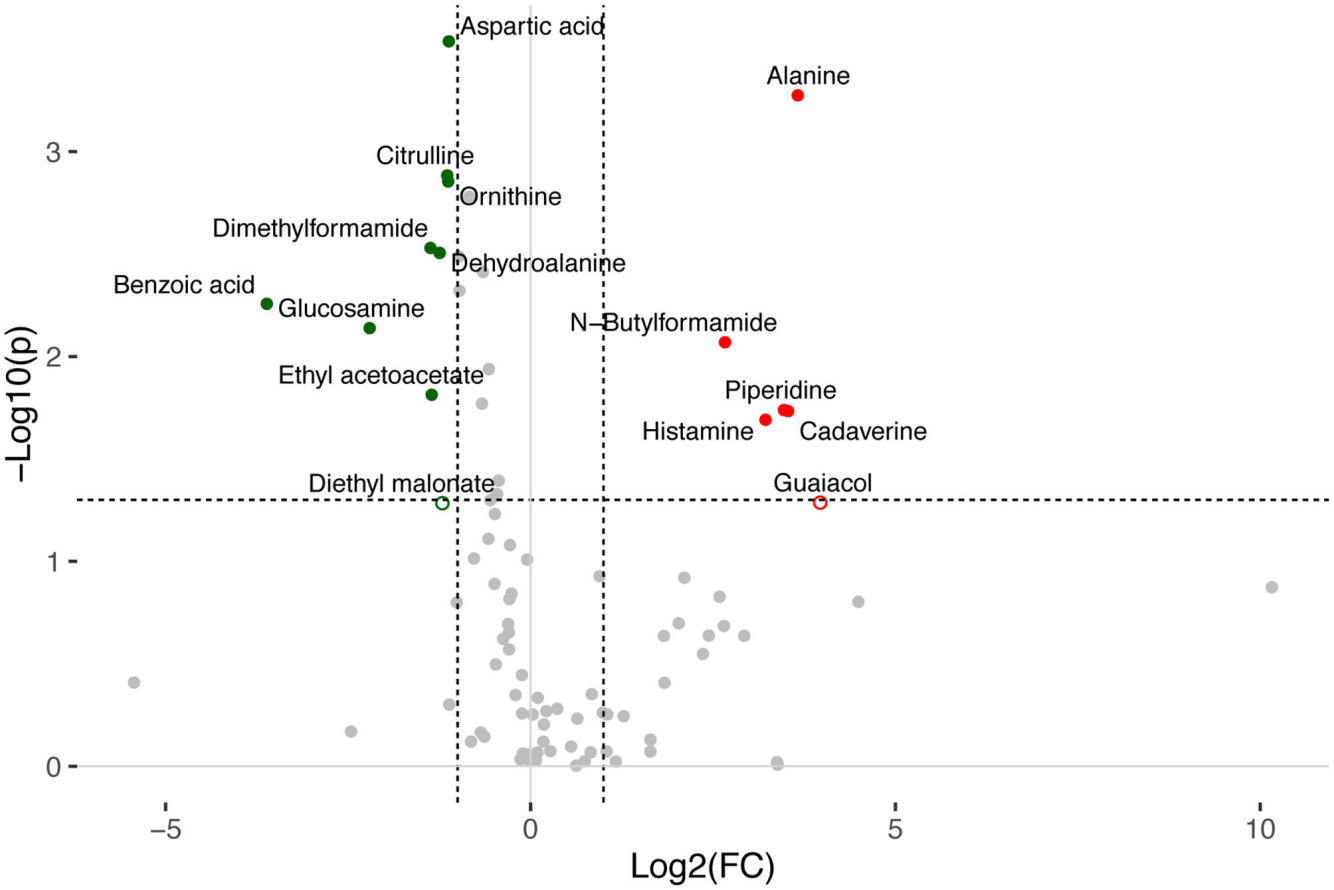
Volcano plot of LC-MS-detected metabolites in healthy and DEC-positive stool samples. Changes in normalized peak intensities of metabolites detected in samples from the DEC group compared to healthy samples. The volcano plot summarizes both fold-change and *t*-test criteria for all metabolites. Metabolites with significant differential levels according to the *t*-test *p*-value (*p* < 0.05) and fold-change value (FC > 2) were colored. Red and green dots represent metabolites found significantly higher and lower, respectively, in the DEC group compared to the healthy group. Borderline metabolites (diethyl malonate and guaiacol) are represented as empty circles.

**Figure 5:**
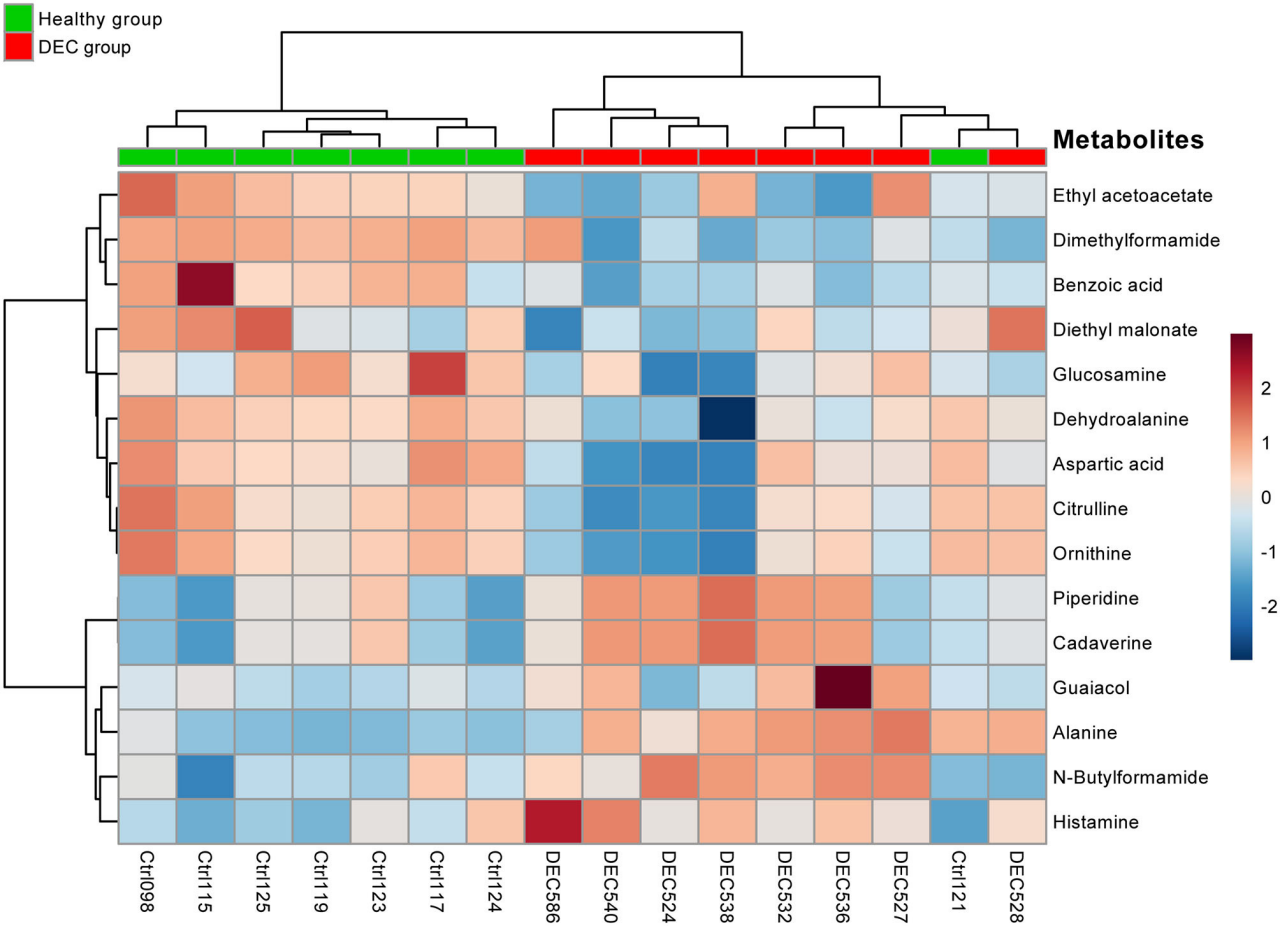
Heatmap of main metabolites found in healthy and DEC-positive stool samples. Heatmap showing the clustering of the 15 main metabolites present in stool samples found in the DEC and healthy groups. Each colored cell on the map corresponds to a metabolite peak intensity. The row displays the metabolites and the column represents the samples (red = elevated; blue = reduced). A distinct pattern of group-associated metabolite ordination was observed. Metabolites and samples were hierarchically clustered by Ward's method and Pearson's distance using the *MetaboAnalyst* package for R.

A total of 156 metabolites were identified in the 16 stool samples included in this work. We discarded molecules that are not metabolized by bacteria, such as sweeteners or food additives. Therefore, a total of 88 metabolites were included in the analysis. The redundancy analysis of normalized data showed a distinctive metabolic composition for each group (Figure 3; *p* = 0.001). Using a univariate analysis of samples by volcano plot, we found 13 metabolites with a fold change >2 and *p*-value of *t*-test <0.05 (Table 2; Figure 4). Alanine, N-butylformamide, piperidine, cadaverine and histamine were significantly associated with the DEC group compared to the healthy group. On the other hand, aspartic acid, ornithine, citrulline, dimethylformamide, dehydroalanine, ethyl acetoacetate, glucosamine and benzoic acid were significantly associated with the healthy group compared to the DEC group. We included guaiacol and diethyl malonate in the DEC and healthy groups in the following analysis since these metabolites where close to significance (fold change >2 and *p* = 0.05). Dendrograms and heatmaps of the significantly associated metabolites described above displayed a hierarchical organization of samples similar to the *a priori* grouping of samples based on health status (Figure 5).

**Table 2:** Changes of peak intensities of the 15 main LC-MS-detected metabolites in DEC-positive stool samples compared to the healthy stool samples.

	**Compounds**	**Fold Change (FC)**	***p***
Metabolites associated to DEC group	Alanine	12.66^*^	0.0005
	N-Butylformamide	6.34^*^	0.0085
	Piperidine	11.09^*^	0.0183
	Cadaverine	11.55^*^	0.0185
	Histamine	9.32^*^	0.0204
	Guaiacol	15.67	0.0517
Metabolites associated to healthy group	Aspartic acid	−2.17^*^	0.0003
	Ornithine	−2.19^*^	0.0014
	Citrulline	−2.21^*^	0.0013
	Dimethylformamide	−2.59^*^	0.0029
	Dehydroalanine	−2.37^*^	0.0031
	Benzoic acid	−12.25^*^	0.0055
	Glucosamine	−4.61^*^	0.0073
	Ethyl acetoacetate	−2.56^*^	0.0154
	Diethyl malonate	−2.31	0.0582

*Metabolites with statistically significant differential levels according to the t-test p-value and fold-change value are indicated as main metabolites for each group*.

* *p < 0.05*.

### Microbiota-Metabolome Correlation in Samples From DEC and Healthy Groups

**Figure 6:**
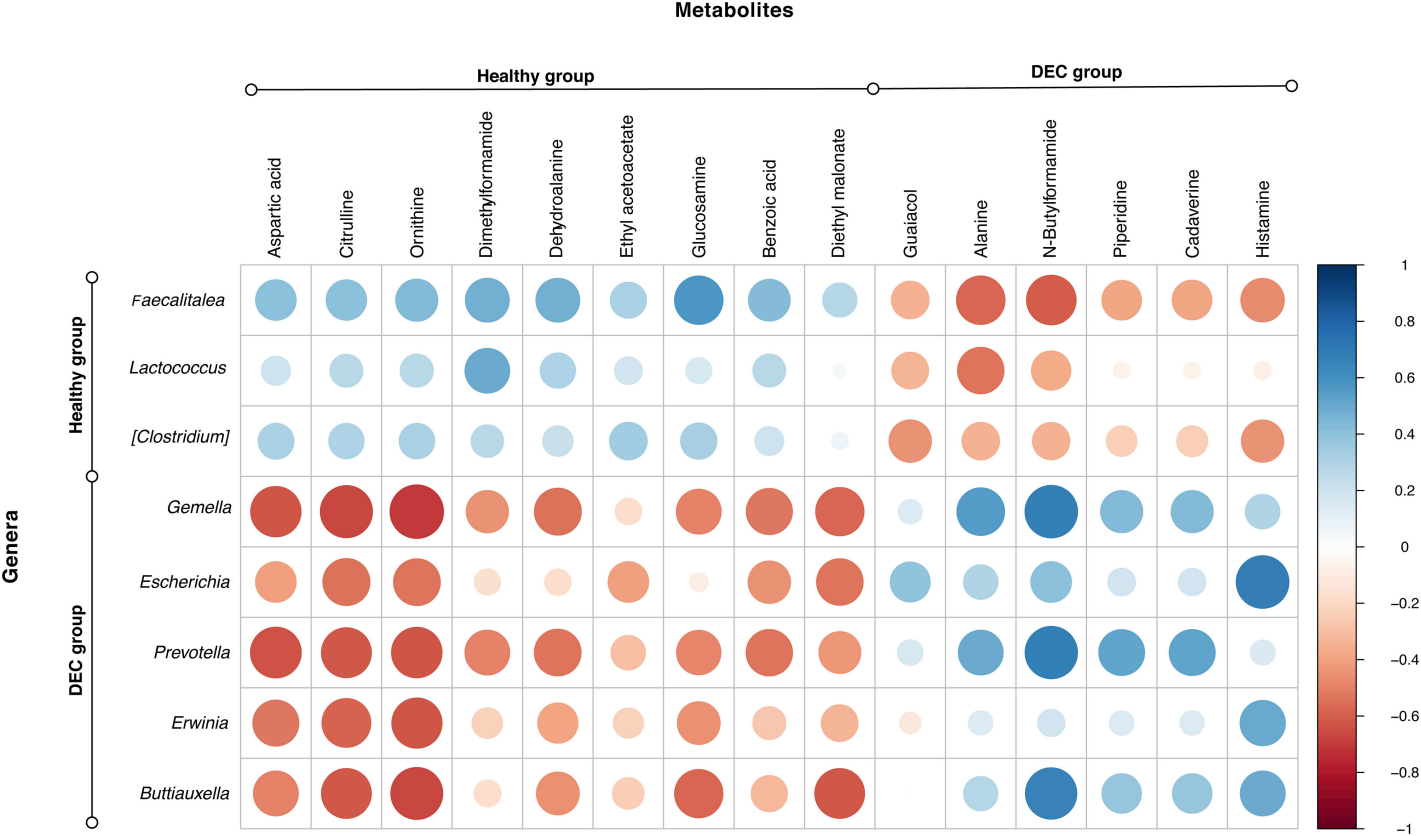
Main genera and metabolites found in healthy and DEC-positive stool samples. Heatmap showing the correlation relationships between peak intensities of the 15 main metabolites, and the abundance of the main genera associated with DEC or healthy groups. Blue and red dots represent positive and negative correlations, respectively, between metabolites and genera. This analysis was done using the *corrplot* package for R.

Among the identified taxa on samples, we found that *Gemella* (*p* = 0.005*), Escherichia (p* = 0.003), *Prevotella* (*p* = 0.027), *Erwinia* (*p* = 0.010), and *Buttiauxella* (*p* = 0.016) were the genera significantly associated with the DEC groups. Instead, *Faecalitalea* (*p* = 0.001)*, Lactococcus* (*p* = 0.032), and *Clostridium* (*p* = 0.010) were the genera significantly associated with the healthy groups. Correlation between the abundance of these main genera and the 15 metabolites with a fold change >2 showed a clear pattern of link between the group's associated genera and metabolites in each group (Figure 6).

**Figure 7:**
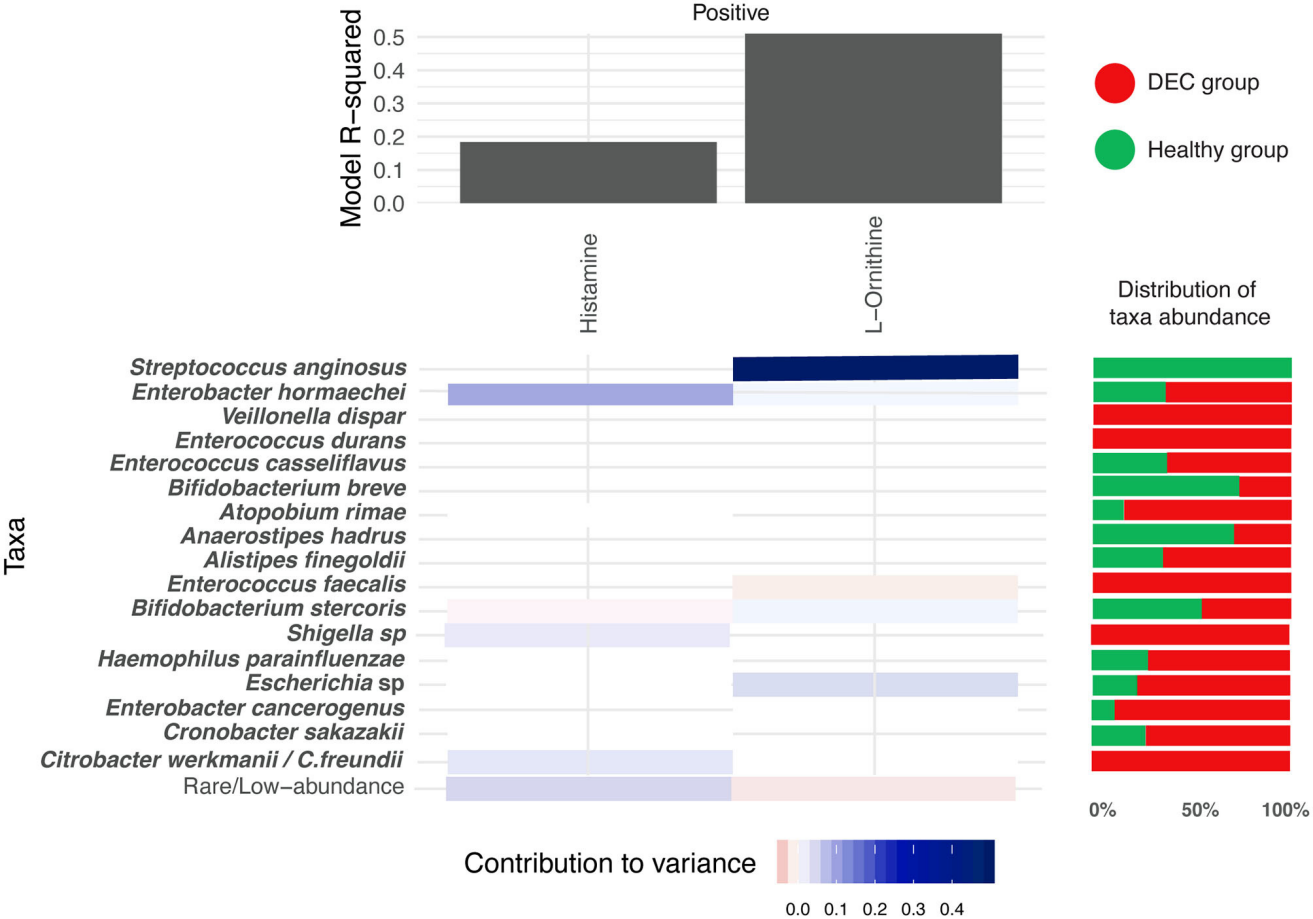
Microbial contribution to metabolite variance. Prediction of microorganisms that positively contribute to variance of the levels for the 15 main metabolites (blue = high contribution; red = low contribution). This analysis was done with the MIMOSA2 software using the microbiome and metabolome data found on each group. R-square of the regression model used for prediction represents the sum of the contributions of all listed taxa to the metabolite variance. Red and green horizontal bars represent the distribution of taxa abundance of proposed organisms among DEC and healthy groups, respectively.

Using the MIMOSA2 software, a tool for the metabolic model-based estimation of paired microbiome and metabolomic datasets, we evaluated the representative microbiota sequences and the abundance of the 15 metabolites described above. We found specific genera and species that could explain the variance of histamine and ornithine, metabolites distinctively found in the DEC and healthy groups. In the case of metabolomic screening for these metabolites, we found higher levels of histamine in the DEC groups compared to healthy groups, which according to the MIMOSA2 model could be explained mainly by the presence of *Enterobacter hormaechei, Bifidobacterium stercoris, Shigella* spp., and *Citrobacter werkmanii/freundii*. We also found lower levels of ornithine in the DEC samples compared to healthy groups (Table 2), which could be due mainly to the presence of *Streptococcus anginosus, Enterococcus faecalis* and *Escherichia* sp. (Figure 7).

## Discussion

Current knowledge of gut microbiota in diarrheal infections reveals a distinctive composition and abundance of commensal bacteria (Jeffery et al., 2019), but these differences are insufficient to explain the mechanisms involved in infections by enteric pathogens. Several reports strongly suggest that DEC pathogenicity along the transit of the pathogen across the gastrointestinal tract is finely regulated by gut microbiota, the immune system and metabolites of the intestinal milieu (Thaiss and Elinav, 2014). Therefore, gut metabolome could provide relevant information to reveal the environmental signals produced by gut microbiota that regulate DEC pathogenesis at a specific time or place (Kumar and Sperandio, 2019).

Our previous evidence has indicated that children with diarrhea by DEC display a distinctive microbiota composition (Gallardo et al., 2017). In this current study we have confirmed our previous observations by analyzing microbiota composition in stool samples from 8 age-paired children, either healthy or with diarrhea by DEC (Figure 1). Considering that dysbiosis could be related to a distinct metabolomic composition (Noorbakhsh et al., 2019), we sought to identify the metabolome composition of the samples included in this study. Interestingly, our data showed a clear metabolomic structure statistically different for each group (Figure 3), suggesting that metabolic environment associated with DEC infection may contain specific metabolites related to DEC pathogenicity. We identified 15 metabolites in stool samples significantly different between the DEC and healthy groups (Figure 4), the levels of which were associated with the main genera from DEC and healthy groups (Figure 5). Considering this well-conserved metabolomic and commensal community structure among the groups, we then sought to identify which metabolic pathways could be involved in what was observed. We found by functional predictions from amplicon sequences that L-histidine biosynthesis and degradation pathways, as well as the L-ornithine biosynthesis route, were significantly different between the DEC and healthy groups (Figure 6). These predictions are in agreement with the low levels of ornithine and histidine detected in the DEC group compared to healthy groups (Table 2; Supplementary Table 1). Interestingly, we found that histamine, a product of L-histidine decarboxylation, was significantly higher in the DEC group compared to the healthy groups (Figure 4). In the gut, histamine is produced by immune cells such as mast cells, and its secretion could be influenced by cytokines such as IL-18, TNF-α, IL-12, and IL-1, among others (O'mahony et al., 2011). In commensal bacteria, such as *E. coli*, histamine has been linked to endogenous biosynthetic pathways (Kyriakidis et al., 2008) in Ca^2+^-mediated signals (Theodorou et al., 2009) and its chemotaxis (Theodorou et al., 2012). The role of histamine could be directly linked to *E. coli* adherence, as evidence has shown that histamine inhibits the clearance of *E. coli* from the host peritoneal cavity in a peritonitis mouse model (Hori et al., 2002). Related to ornithine, we found lower levels of this metabolite in the DEC samples than in the healthy ones. L-ornithine can be produced in the urea cycle by the enzyme arginase, using arginine as the substrate. Once produced, L-ornithine is transformed to citrulline by the enzyme ornithine carbamoyltransferase (Wu, 1998). Arginine levels were similar in both groups, but citrulline and ornithine levels were higher in healthy samples (Table 2). L-ornithine production has been associated with a healthy gut mucosa. A recent study showed that L-ornithine administered to mice resulted in goblet cell production, mucin secretion and cell proliferation, which are associated with a healthier gut environment (Qi et al., 2019). Together with these results, L-ornithine also induces accumulation of IL-22 in intestinal tissues (Qi et al., 2019), a cytokine involved in the reconstitution of gut epithelial cells, improving mucus production by goblet cells, increasing the production of antimicrobial peptides and modulating genes involved in wound healing (Sun et al., 2012). Overall, these observations suggest that histamine and L-ornithine might be important signaling molecules in DEC pathogenesis and merit further investigation.

MIMOSA2 modeling of the 15 metabolites statistically associated with the study groups suggested that only 2 metabolite (ornithine and histamine) variations depend on the presence of specific taxa. Among the taxa associated with the positive contribution of histamine and ornithine levels, *Escherichia* sp., *Streptococcus anginosus, Citrobacter werkmanii/Citrobacter freundii*, and other species were found to increase the variability of these metabolites (Figure 7). Within these genera, *Escherichia coli* has been identified as being responsible for histamine production (Barcik et al., 2016, 2017), suggesting that during DEC infection, histamine levels could be modulated by pathogen metabolism. For the other 13 metabolites, the metabolic potential scores of identified taxa were not predictive according to the MIMOSA2, meaning that metabolite levels could not be attributed to specific taxa of the DEC or the healthy group. However, piperidine and cadaverine have been associated with virulence in enteropathogens. A decrease in *S. flexneri* invasion to cells was found in the presence of ornithine; this effect was found to be counteracted by the presence of cadaverine, a polyamine produced by lysine decarboxylation (Durand and Bjork, 2009). In the intestine, cadaverine is cyclated to piperidine, a molecule that reduces the invasion of *S. typhimurium* and the recruitment of polymorphonuclear neutrophils (Kohler et al., 2002). It would be interesting to evaluate the role that piperidine and cadaverine might play in the virulence of DEC, as well as the other metabolites significantly associated with the DEC group.

Our study has limitations. We analyzed a small number of samples and therefore our results should be considered as an exploratory approach. However, our data provided valuable information about the importance of gut microbiota and metabolome analysis in understanding the regulatory mechanism of DEC virulence. It is important to note that our metabolome analysis did not include metabolites that have been previously proven to have an impact on DEC virulence, such as SCFAs, because the primary goal of this study was to identify new pathways or metabolites that might be important to DEC pathogenicity. From this study we obtained an effect size value of 0.4377, which will be used to obtain the required sample size for further studies to confirm our observations, as well as *in vitro* infection experiments to evaluate the role of metabolites significantly associated with the DEC group as signaling molecules involved in DEC virulence regulation.

In conclusion, our study showed that stool samples from healthy children and children positive for DEC displayed a differential metabolome and microbiota composition. We found a correlation between gut microbiota species and certain metabolites, information that might be useful in identifying mechanisms and signaling molecules implicated in the crosstalk between microbiota and DEC pathotypes that might participate in the virulence of these pathogens.

## Data Availability Statement

The datasets presented in this study can be found in online repositories. The names of the repository/repositories and accession number(s) can be found below: https://www.ncbi.nlm.nih.gov/, PRJNA623942.

## Ethics Statement

The studies involving human participants were reviewed and approved by Ethical Committee of Universidad de Chile. Written informed consent to participate in this study was provided by the participants' legal guardian/next of kin.

## Author Contributions

PG participated in sample processing, performed sequences and metabolites analyses, interpreted the data, and participated in manuscript writing. MI participated in data analysis and manuscript writing. RV participated in study design and data analysis. FS participated in data analysis and manuscript writing. JO participated in data analysis. MF participated in study design, data acquisition and interpretation, manuscript writing, and final approval of the manuscript. All authors contributed to the article and approved the submitted version.

## Conflict of Interest

The authors declare that the research was conducted in the absence of any commercial or financial relationships that could be construed as a potential conflict of interest.
