# Supplementary material for: Gut Microbiota-Metabolome Changes in Children With Diarrhea by Diarrheagenic E. coli
Source: Front Cell Infect Microbiol. 2020 Sep 18;10:485. doi: 10.3389/fcimb.2020.00485 (PMC7531578; doi:10.3389/fcimb.2020.00485)
Supplement: Supplementary file 1 [file Data_Sheet_1.pdf]

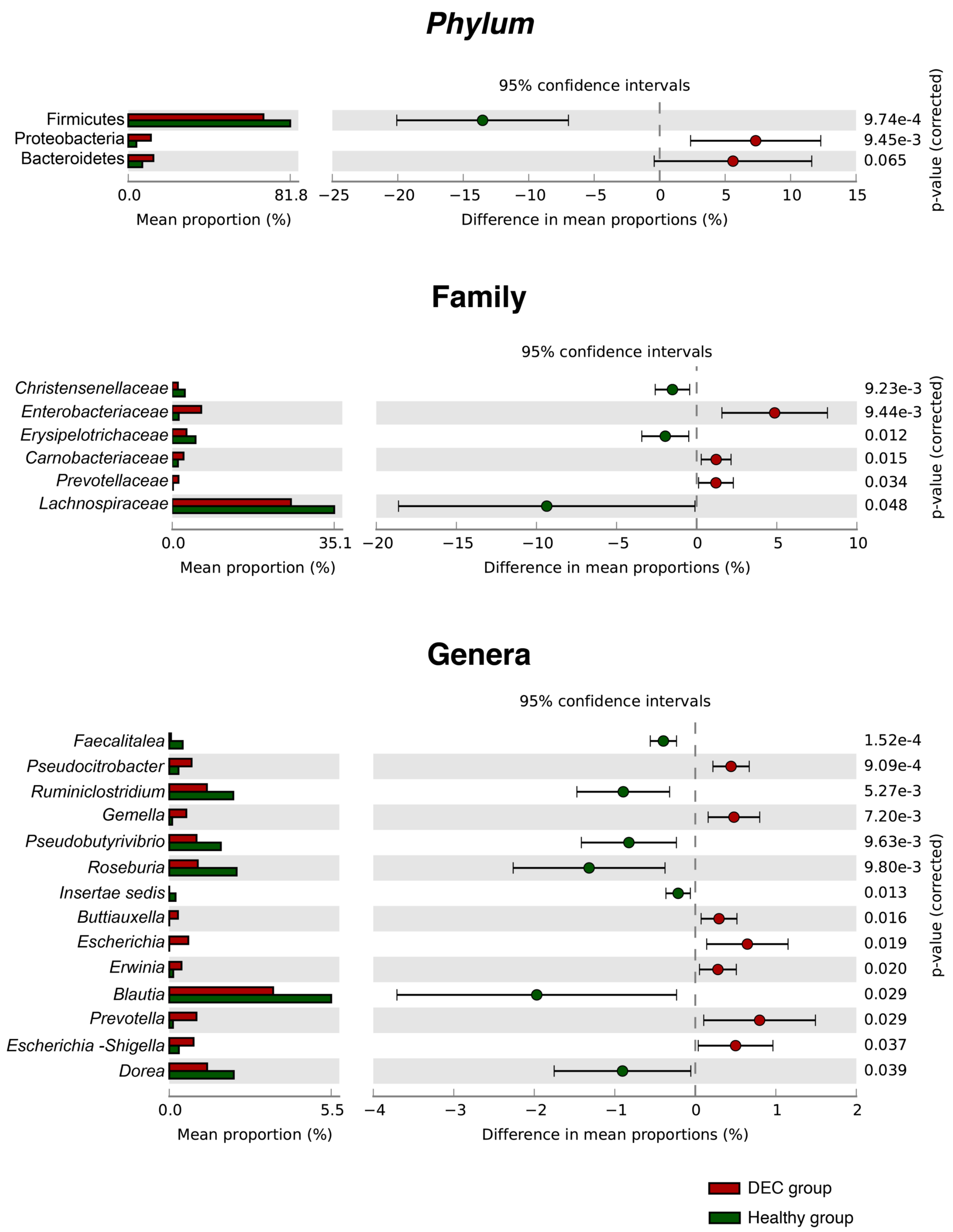

Supplementary Figure 1. Significant taxa found in healthy and DEC-positive stool samples. Relative abundance of the significant taxa at *Phylum*, family and genus level for DEC and healthy groups. STAMP software was used to calculate the taxa proportions based on an OPU determination. Welch's t-test was used to compare abundance between the two groups.

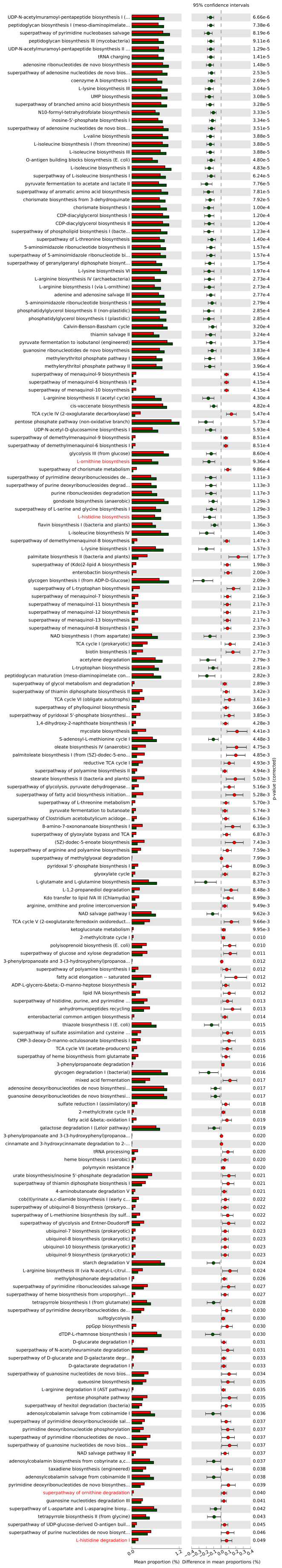

Supplementary Figure 2. Predicted metabolic pathways found in healthy and DEC-positive stool samples. Barr plot of significant predicted pathways by PICRUSt2 software using 16S rRNA gene sequences found in DEC and healthy groups. This prediction was done using representative sequences of main OPUs from each group and the Greengenes database as the reference. Prediction graphs were obtained using STAMP v. 2.1.3 and the significance value was determined with Welch's t-test. Pathways involved in L-ornithine and L-histidine metabolism are highlighted in red.

Supplementary Table 1. Changes of peak intensities of the LC-MS-detected metabolites in the DEC-positive stool samples compared to the healthy samples. Fold changes (FC) and t-test p-value of metabolite levels in the DEC group compared to the healthy samples are shown.

Metabolite	FC	pValue
Isoleucine	-1.79	0.0017
Gamma-Aminobutyric acid	-1.98	0.0033
Methionine	-1.57	0.0039
Glutamic acid	-1.96	0.0048
Serine	-1.49	0.0115
Glycine	-1.59	0.0170
Lysine	-1.35	0.0404
Valine	-1.37	0.0469
Threonine	-1.46	0.0502
Tyrosine	-1.40	0.0587
N-Acetyl-L-leucine	-1.49	0.0776
Histidine	-1.22	0.0833
N-Acetyl-DL-methionine	-1.71	0.0968
Dodecanedioic acid	-1.03	0.0980
Phosphoric acid	1.93	0.1181
Carbofuran	4.31	0.1204
Thymine	-1.41	0.1288
Sorbitol/manitol	3.94	0.1338
Phenylalanine	-1.20	0.1436
Succinic acid	6.02	0.1489
Leucine	-1.22	0.1526
DL-Mevalonic acid	22.50	0.1575
E-Ferulic acid	-2.02	0.1588
3-Phenylpropanoic acid	4.09	0.2006
Uracil	-1.24	0.2022
Succinic anhydride	6.28	0.2068
Xanthine	-1.23	0.2224
Propionic acid	5.44	0.2304
E-p-coumaric acid	7.61	0.2307
Paraldehyde	3.55	0.2312
Glutaric acid	-1.30	0.2389
Neuraminic acid	-1.23	0.2691
Kynurenic acid	5.14	0.2832
Methionine S-oxide	-1.39	0.3184
Tryptophan	-1.08	0.3590
Leucyltyrosine	-43.24	0.3904

3,3'-1,4-Butanediyldiiminodipropanoic acid	3.57	0.3914
N-Acetyl-L-phenylalanine	1.79	0.4446
L-Theanine	-1.15	0.4489
8-Amino-7-oxononanoic acid	1.07	0.4632
Vitamin C	-2.17	0.4998
Urocanic acid	1.29	0.5240
Gly-Leu	1.16	0.5385
4-Nitrophenol	1.99	0.5477
Isopelletierine	-1.08	0.5526
Inosine	2.07	0.5588
D-Gluconic acid	1.02	0.5593
Pyruvic acid	2.42	0.5701
N-Acetylvaline	1.56	0.5854
threonic acid	1.14	0.6255
Sinapyl alcohol	-5.50	0.6768
Phenylethyl alcohol	-1.60	0.6837
Desaminotyrosine	-1.55	0.7167
3-hydroxybutyric acid	3.12	0.7421
Adipic acid	-1.76	0.7578
Asparagine	1.13	0.7589
methylxanthine	1.47	0.8014
Proline	1.21	0.8436
5-Hydroxyindoleacetate	2.06	0.8455
Uric Acid	3.12	0.8472
N6,N6,N6-Trimethyl-L-lysine	1.77	0.8547
Hexose	1.07	0.8572
Taurine	-1.08	0.8665
Malondialdehyde	-1.02	0.8764
Suberic acid	-1.06	0.9019
L--Erythrulose	-1.10	0.9243
Lactic acid	1.05	0.9361
Diethylpyrocarbonate	-1.01	0.9425
Arginine	1.67	0.9455
Pyroglutamic acid	2.25	0.9490
Glyceraldehyde	10.38	0.9540
Traumatic Acid	10.46	0.9826
Pantothenic acid	1.54	0.9928

Supplementary Table 2. Normalized peak intensities of main 15 LC-MS-detected metabolites found in this study. Sample normalization of peak intensities was done by median and data log transformed without scaling.

Sample	Group	**Ethyl acetoacetate**	**dimethylformamide**	**Benzoic acid**	**Diethyl malonate**	**Glucosamine**	**dehydroalanine**	**Aspartic acid**	**Citrulline**	**Ornithine**	**Piperidine**	**Cadaverine**	**Guaiacol**	**Alanine**	**N-Butylformamide**	**Histamine**
**Ctrl098**	Healthy	4.705	-0.531	2.876	2.182	-0.380	0.335	4.286	2.717	0.334	-2.387	-3.506	-6.049	-1.041	-2.844	-2.060
**Ctrl115**	Healthy	4.749	0.653	6.899	3.600	-0.314	0.810	4.697	3.358	0.921	-2.714	-3.841	-4.589	-4.931	-5.259	-2.289
**Ctrl117**	Healthy	2.972	-0.003	2.957	-0.679	3.248	0.400	4.619	2.448	0.132	-1.301	-2.431	-5.506	-4.883	-1.283	-1.399
**Ctrl119**	Healthy	3.371	-0.344	2.542	0.796	1.935	0.051	4.126	2.147	-0.186	1.606	0.309	-6.600	-6.177	-3.202	-2.490
**Ctrl121**	Healthy	1.777	-3.191	1.016	0.870	-0.903	0.008	4.203	2.323	0.062	-0.065	-1.286	-5.932	4.141	-4.458	-3.245
**Ctrl123**	Healthy	3.501	0.179	3.407	0.906	0.535	0.232	4.138	2.745	0.378	3.543	2.343	-6.020	-5.636	-3.366	-0.103
**Ctrl124**	Healthy	2.686	-0.281	0.925	1.970	1.065	0.339	4.731	2.479	0.168	-2.744	-3.857	-6.236	-5.331	-2.831	0.847
**Ctrl125**	Healthy	3.589	-0.138	2.155	3.838	1.361	-0.012	4.001	2.078	-0.173	1.345	0.127	-6.167	-5.736	-3.225	-2.032
**DEC524**	DEC	1.927	-1.930	1.325	-0.053	-2.675	-0.668	3.279	1.597	-0.814	5.827	4.698	-6.408	1.883	1.656	0.713
**DEC527**	DEC	2.887	-3.950	-1.242	-1.388	-0.659	-1.933	2.111	-0.041	-2.530	-2.733	-4.000	-4.377	5.760	-1.540	-1.975
**DEC528**	DEC	2.097	-4.465	0.860	3.547	-1.568	-0.425	3.715	2.529	0.221	1.152	-0.051	-6.056	4.717	-4.384	0.052
**DEC532**	DEC	-0.831	-4.902	0.273	0.567	-1.511	-1.488	3.343	1.083	-1.341	3.673	2.425	-4.344	4.790	-1.485	-1.434
**DEC536**	DEC	-1.277	-5.001	-1.250	-0.827	-0.711	-1.816	3.041	1.402	-0.802	3.735	2.585	0.959	5.574	-0.605	-0.038
**DEC538**	DEC	4.387	-4.363	0.598	-0.536	-3.228	-3.851	2.475	0.653	-1.804	6.361	5.219	-5.696	5.229	0.337	1.456
**DEC540**	DEC	0.428	-4.836	-0.646	0.696	0.919	-1.325	2.813	0.816	-1.354	5.276	4.072	-2.694	5.094	-1.601	2.515
**DEC586**	DEC	1.059	1.133	2.164	-1.569	-0.773	0.477	4.192	1.997	-0.352	2.526	1.361	-3.728	-3.283	-0.594	4.660
